# Return to sport after primary anterior cruciate ligament reconstruction in athletes with a preinjury Tegner Activity Scale ≥7: a longitudinal, nationwide, registry-based cohort study

**DOI:** 10.1136/bjsports-2025-111497

**Published:** 2026-10-08

**Authors:** Daniel Castellanos Dolk, Henrik Hedevik, Per Wretenberg, Anders Stålman, Joanna Kvist

**Affiliations:** 1 Department of Molecular Medicine and Surgery, Stockholm Sports Trauma Research Center, Karolinska Institute, Stockholm, Sweden; 2 Department of Orthopedic Surgery, Capio Specialistvård Motala, Motala, Sweden; 3 Physiotherapy, Linkoping University Department of Health Medicine and Caring Sciences, Linköping, Sweden; 4 Örebro University School of Medical Sciences, Orebro, Sweden; 5 Department of Orthopedics, Örebro University Hospital, Orebro, Sweden; 6 Capio Artro Clinic, FIFA Medical Centre of Excellence, Sophiahemmet Hospital, Stockholm, Sweden

**Keywords:** Anterior Cruciate Ligament, Sports medicine, Women in sport, Athletes, Knee injuries

## Abstract

**Objective:**

This study aimed to longitudinally assess the rate and timing of return to sports (RTS) and its predictors after primary anterior cruciate ligament reconstruction (ACL-R) in preinjury Tegner Activity Scale≥7 (TAS≥7) athletes.

**Methods:**

This prospective, longitudinal, registry-based cohort study included 511 athletes aged 16–40 with a preinjury TAS≥7 registered in the Swedish National Knee Ligament Registry following primary ACL-R. The primary outcomes were RTS TAS≥7 and associated predictors. Secondary outcomes included RTS at preinjury TAS levels, associated predictors, secondary ACL injury rates and assessment of patient-reported outcome measures (PROMs). Associations were analysed using log-binomial and multivariable Cox regression.

**Results:**

62% (n=315) achieved RTS TAS≥7 after ACL-R within 12.3 months (SD 4.7), while only 51% achieved RTS preinjury TAS level within 12.7 months (SD 4.9). Negative predictors for RTS TAS≥7 included female sex, older age, low sporting level and delayed surgery. ACL-Return to Sports after Injury (ACL-RSI) score >4.2 at 3 months postoperatively was associated with higher likelihood of RTS TAS≥7 and RTS preinjury TAS level. No differences in secondary ACL injury rates were observed between RTS groups or sexes, with overall secondary ACL injury rates of 9.2% (n=47) within 24 months. Participants returning to TAS≥7 reported better PROMs.

**Conclusion:**

Three out of five athletes returned to knee-demanding sports following ACL-R. Female sex, older age, lower sporting level and delayed surgery were associated with reduced RTS≥7 rates. Psychological readiness at 3 months post-surgery emerged as a potentially modifiable factor associated with RTS outcomes after ACL-R.

**WHAT IS ALREADY KNOWN ON THIS TOPIC**

- Return to sport (RTS) after anterior cruciate ligament reconstruction (ACL-R) remains a key outcome for athletes, yet many do not resume preinjury levels of activity, and although prior research has largely been retrospective or limited to a single athletic level or sex, despite consistent findings that female athletes tend to have lower RTS rates than males.

**WHAT THIS STUDY ADDS**

- This prospective study of 511 female and male athletes undergoing primary ACL-R with a Tegner Activity Scale score of≥7 (TAS ≥7) before ACL injury provides monthly follow-up data that adds to the knowledge of RTS and its predictors.
- The athletes involved in the present study achieved RTS TAS≥7 within 24 months in 62% of cases, with an average duration of 12.3 months (SD 4.7), and with higher patient-reported outcome measures. Nonetheless, only 51% of the participants returned to their preinjury level of sports participation with a mean time of 12.7 months (SD 4.9), indicating the persistent challenge of fully recovering after ACL reconstruction.
- Female sex, older age, lower preinjury sporting level and delayed surgery (>12 months post-ACL injury) were associated with reduced likelihood of RTS TAS≥7. In comparison, psychological readiness at 3 months (ACL-Return to Sports after Injury score (ACL-RSI)≥4.2) was a strong, potentially modifiable factor associated with successful RTS TAS≥7 after ACL-R.

**HOW THIS STUDY MIGHT AFFECT RESEARCH, PRACTICE, OR POLICY**

- This study emphasises the importance of prospective, longitudinal follow-up of athletes after ACL-R and supports the use of ACL-RSI as a tool to identify individuals at risk of not returning to sport, while observed disparities across sex, age and sporting level underscore the need for targeted, inclusive rehabilitation strategies and further research into the mechanisms underlying these differences.

## Introduction

 Athletes participating in pivoting sports are at high risk of anterior cruciate ligament (ACL) rupture, an injury that can significantly affect their sports participation and career continuation.^1^ The heightened exposure to cutting and pivoting situations in these sports contributes to high injury incidence rates, particularly among female athletes, who have a 3.3-fold higher incidence of ACL reconstruction (ACL-R) compared with male athletes.^2 3^

Although ACL-R is commonly performed with the aim of facilitating return to sports (RTS), achieving successful RTS is not guaranteed.^4 5^ Approximately one quarter of athletes do not RTS after ACL-R, with both knee-related and psychological factors, such as fear of reinjury, influencing outcomes.^6^ In addition, female athletes demonstrate lower RTS rates and poorer functional and subjective outcomes after ACL-R compared with their male counterparts.^7^

Interpretation of RTS outcomes is further complicated by substantial variability in how RTS is defined across studies. RTS has been described as a continuum, encompassing return to participation, return to sport and return to performance.^8^ However, several studies collapse these different stages of RTS into a single RTS endpoint and instead define RTS as the athlete participating in a training or competition, limiting insight into the RTS process and complicating the comparisons between studies.^9^

In Sweden, individuals undergoing ACL-R have been thoroughly studied throughout the years using a high-quality registry with over 90% coverage, the Swedish National Knee Ligament Registry (SNKLR).^10^ However, the SNKLR does not include detailed information on the individual’s prior level of sports participation or their RTS outcomes, and studies focusing on RTS using registry data outside of specific cohorts remain limited.

Therefore, the aim of this study was to longitudinally follow male and female athletes undergoing primary ACL-R who were participating in highly knee-demanding sports, defined as a preinjury Tegner Activity Scale of ≥7 (TAS ≥7). Athletes were followed for 24 months, and RTS was defined based on achieving an activity level of TAS≥7 together with the level of participation in sport, allowing distinction between full and reduced return to sports. The primary objective was to: (1) determine the rate and timing of RTS TAS≥7 and identify factors associated with successful RTS TAS≥7. In addition, we aimed (2) to evaluate the rate and timing of return to participation and RTS preinjury TAS level, (3) the factors associated with achieving RTS preinjury TAS level, (4) compare secondary ACL injury rates across sexes and RTS TAS≥7 status and (5) describe patient-reported outcome measures (PROMs) by RTS TAS≥7 status.

## Material and methods

### Study sample

The SPARX (Sports and Physical Activity after Reconstruction of the Anterior Cruciate Ligament[X]) study is a longitudinal, prospective, national registry-based cohort study. Patients in the current cohort were prospectively recruited from 25 November 2019 to 31 May 2021 following ACL-R registration in the SNKLR.

Inclusion criteria were primary ACL-R, age 16–40 years at surgery, preinjury TAS≥7, fluency in Swedish, good cognitive function and an available mobile phone or email for the research team to send digital surveys. The age range of 16–40 years was selected to include skeletally mature athletes while focusing on a population for whom return to high-level sport after ACL reconstruction is most clinically relevant. In the present study, a preinjury TAS≥7 was used to define participation in highly knee-demanding sports, consistent with prior work in which TAS levels 7–10 represent competitive sports involving high-speed actions and frequent pivoting movements.^11^

Exclusion criteria were contralateral ACL injury, revision ACL-R and multiligament knee injury surgery (defined as surgery of two or more ligaments), and concomitant fractures and/or vascular injuries. Participants with missing data for the relevant variables from SNKLR or the study surveys were also excluded from the analyses. Individuals participating in other studies of importance that could either affect the outcome or adherence to the current study were also not eligible.

Recruitment was conducted within 3 months post-ACL-R, requiring registration in the SNKLR within 1.5 months after surgery to allow the study team to identify and contact potential participants. All potential participants were pseudo-anonymised prior to recruitment and received an individual ID code to be used during recruitment, the planned follow-up, and data analysis.

An information letter introducing the study was sent to eligible patients’ home addresses, along with a QR code to scan, leading to a registration webpage where they could provide their mobile phone number and/or email if they wanted to participate. Patients who did not respond via the QR code but had a registered mobile phone number in the SNKLR were contacted by telephone. Patients who expressed interest in participating received a final invitation to be included in the study via email or SMS. As shown in the flowchart (figure 1), 80 participants were lost to follow-up at various intervals before a potential RTS assessment could be conducted within 24 months after ACL-R, resulting in a survey response rate of 84% at 24 months (and 94% at 12 months).

**Figure 1:**
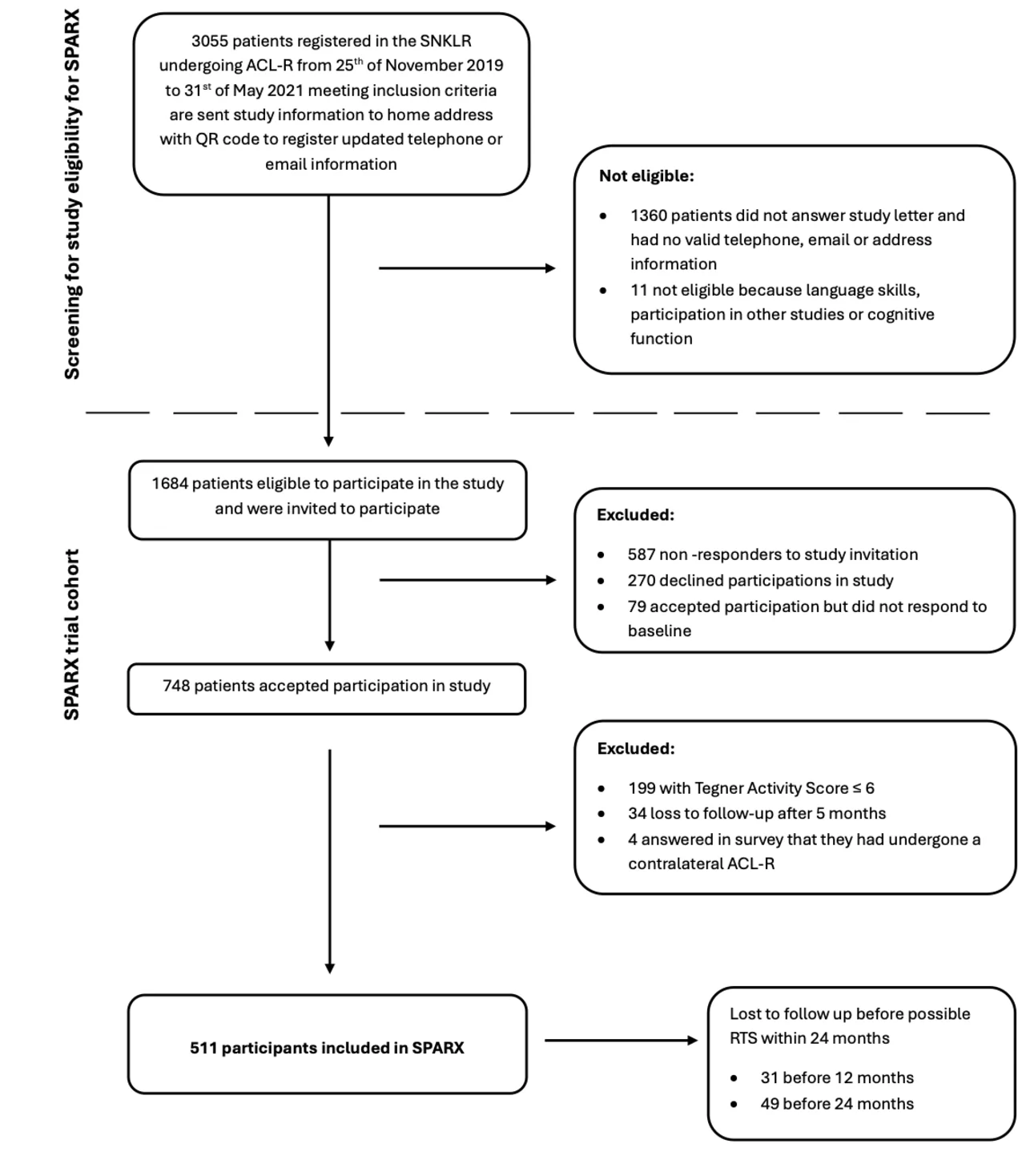
Flowchart of patient screening, exclusion and inclusion of cohort. ACL-R, anterior cruciate ligament reconstruction; RTS, return to sports; SNKLR, Swedish National Knee Ligament Registry; SPARX, Sports and Physical Activity after Reconstruction of the Anterior Cruciate Ligament[X].

This study adheres to the STROBE (Strengthening the Reporting of Observational Studies in Epidemiology)^12^ and RECORD (REporting of studies Conducted using Observational Routinely collected health Data)^13^ guidelines for reporting registry-based cohort studies.

### Data collection

Study participants were sent web-based surveys monthly during the first 2 years after undergoing primary ACL-R. The monthly surveys, starting at 3 months (baseline), covered topics such as RTS questions regarding participation in rehabilitation and sports, knee function and new injuries. Advanced questionnaires were administered at baseline (3 months), 6 months, 12 months and 24 months, including validated questionnaires to assess PROMs. Demographic and surgical data were retrieved from the SNKLR, and patients reporting second ACL injuries were double-checked in the SNKLR. Reminder surveys were sent by SMS or email for all monthly surveys; individuals who did not respond to the reminder surveys at 12 and 24 months were contacted by telephone to complete RTS questions.

### Outcome measures

The primary outcome measures were the rate and time to RTS TAS≥7 within 24 months of follow-up. Patient characteristics (age, sex, associated injuries, surgical characteristics) and ACL-Return to Sports after Injury score (ACL-RSI) at 3 months were included as predictors of RTS TAS≥7 using survival analysis.

Secondary outcome measures included: (1) the rate and time to return to participation and RTS preinjury TAS level within 24 months; (2) survival analysis of patient factors that could potentially predict RTS preinjury TAS level, (3) secondary ACL injury rates within 24 months and (4) PROMs at 12- and 24 months.

Assessment of the activity level for RTS was conducted by classifying participants’ responses regarding physical activity or sport and then assigning the TAS level they were participating in according to the modified version of the TAS.^11 14^ The TAS can be used to assess preinjury and postoperative activity levels using a grading system from 0 to 10, where 10 represents the most knee-demanding sports or activities.

Assessment of psychological readiness for RTS was performed using the Swedish version of ACL-RSI,^15^ which uses a 1–10 Likert scale to grade answers and is a well-validated and reliable ACL-specific 12-item tool used to cover important psychological aspects of RTS (eg, emotions, confidence in performance and risk appraisal in relation to RTS).

Evaluation of patient-reported knee function was performed monthly using the Single Assessment Numeric Evaluations^16^ and at the advanced questionnaires using the Swedish version of the International Knee Documentation Committee – subjective Knee Form,^17^ both with a score of 0 to 100 points.

Assessment of knee problems following ACL-R during rehabilitation and participation in sports was measured monthly using the Oslo Sports Trauma Research Center (OSTRC) overuse injury questionnaire,^18^ a validated tool developed to follow athletes and (overuse) injuries over time. This questionnaire consists of four questions regarding sports participation, modified training, performance and pain. For the current study, we used the first question from the OSTRC questionnaire (‘Have you had any difficulties participating in normal training and competition due to knee problems during the past week?’) as part of the RTS definition.

### Return to sports definition

RTS was defined in three levels depending on the participants’ answer to the first question from the OSTRC questionnaire:

1. Return to participation: Reduced participation due to knee problems in a TAS≥7 sporting activity.
2. RTS TAS≥7 level: Full participation with or without knee problems in a TAS≥7 sporting activity (primary outcome).
3. RTS preinjury TAS level: Full participation with or without knee problems at the preinjury TAS level or higher.

### Equity, diversity and inclusion statement

No specific considerations were undertaken during the planning and design regarding equity, diversity and inclusion principles. Our study, however, included individuals registered in the SNKLR regardless of ethnic origin, culture, educational level or socioeconomic background. Sex was accounted for, and female and male athletes were analysed separately and compared. The author group consists of one woman and four men, with different backgrounds and with a different range of experience clinically and in research (ie, three orthopaedic surgeons, one statistician, one physical therapist).

### Statistical analyses

Statistical analyses were performed using SPSS Statistics V.29 (IBM Corp., Armonk, New York, USA). Data analysis and presentation were consistent with the CHecklist for statistical Assessment of Medical Papers (CHAMP) statement.^19^ Non-response analysis was conducted to assess the distribution of differences between study participants (n=748) and the non-included individuals (n=936). The normal distribution was assessed for all continuous variables, and the Student’s test and Mann-Whitney U test were used accordingly. Continuous variables were expressed as means with SD or median with IQR. The χ^2^ test was used for categorical variables, presented as frequencies and percentages. Log-binomial regression was used to estimate relative risk ratios between sexes and sporting level for RTS, and to estimate the relative risk of secondary ACL injuries between sexes and RTS TAS≥7 status.

Survival analyses on time to RTS were performed using R Statistical Software (V.4.4.1; R Core Team 2021). R package ggsurvfit (V.1.0.0) was used to create Kaplan-Meier curves. Censoring was considered for all cases at the time point lost to follow-up before possible RTS TAS≥7 at 24 months. Participants with second ACL injuries (n=13) or pregnancies (n=3) prior to RTS were also censored, as these events prevented further eligibility for RTS in the context of this analysis. Univariate Cox proportional hazards regression analysis using R package survival (V.3.8-3) identified potential predictors of RTS TAS≥7 and RTS preinjury TAS level (online supplemental table 4). The final multivariable models were adjusted for significant predictors of the primary outcome identified in the univariable analysis, in addition to sex and the presence of associated injuries, which were considered clinically significant. Associated injuries and their corresponding surgical procedures were not included simultaneously because variance inflation factor assessment indicated moderate collinearity among meniscus-related variables. Therefore, associated injury variables were retained in the primary analyses because they represent the underlying pathology of interest.

The multivariable model was conducted using complete-case analysis (n=490), with 21 observations excluded due to missing ACL-RSI at 3 months. Baseline characteristics (sex, age, preinjury Tegner level and sporting level) of individuals with and without missing ACL-RSI data were compared to assess potential differences between included and excluded participants.

The results of the regression analyses are reported as HRs with 95% CIs, and a p value of less than 0.05 was considered statistically significant in all analyses. The time from injury to ACL-R was classified into four categories (0–3 months, 3–6 months, 6–12 months and >12 months), similar to that proposed by Cristiani *et al*, to facilitate a parallel to clinical practice.^20^ The ACL-RSI score was dichotomised for the Cox regression analysis using the lowest cut-off reported in the literature (ACL-RSI>4.2)^21^ to facilitate clinical interpretation and comparison with existing literature.

## Results

### Patient demographics

A total of 232 males and 279 females with a preinjury TAS level≥7 were included. Table 1 shows the demographics and patient characteristics of the study participants, stratified by gender. Football was the most predominant sport that athletes participated in before the injury, followed by floorball and handball. Males were more frequently involved in football (60% vs 51%; p<0.001) and females more often in handball (13% vs 6%; p<0.001).

**Table 1:** Patient demographics and surgical characteristics

	Male (n=232)	Female (n=279)	Total (n=511)	P value
**Patient demographics**				
Age (years), mean (SD)	25.6 (6.8)	22.6 (5.9)	23.9 (6.5)	<0.001*
Age categories, n (%)				<0.001
16–19 years	66 (28)	127 (46)	193 (38)	
20–24 years	57 (25)	71 (25)	128 (25)	
25–29 years	45 (19)	46 (16)	91 (18)	
≥30 years	64 (28)	35 (13)	99 (19)	
Sporting level prior to ACL injury, n (%)				0.015
Elite	53 (23)	85 (31)	138 (27)	
Competitive	149 (64)	176 (63)	325 (64)	
Recreational	30 (13)	18 (6)	45 (9)	
Tegner Activity Scale prior to ACL injury, n (%)				0.572
Tegner 10	28 (12)	37 (13)	65 (13)	
Tegner 9	110 (47.5)	116 (42)	226 (44)	
Tegner 8	45 (19.5)	56 (20)	101 (20)	
Tegner 7	49 (21)	70 (25)	119 (23)	
Sports activity prior to ACL injury, n (%)				0.006
Football	138 (60)	143 (51)	281 (55)	
Floorball	38 (16)	42 (15)	80 (16)	
Handball	13 (6)	35 (13)	48 (9)	
Martial arts	9 (4)	12 (4)	21 (4)	
Alpine skiing	7 (3)	11 (4)	18 (4)	
Basketball	6 (3)	9 (3)	15 (3)	
American football/rugby	3 (1)	7 (3)	10 (2)	
Ice hockey	1 (0)	5 (2)	6 (1)	
Other sports	17 (7)	15 (5)	32 (6)	
Associated injuries, n (%)
Medial meniscus injury	82 (35)	72 (26)	154 (30)	0.019
Lateral meniscus injury	97 (42)	90 (32)	187 (37)	0.026
Cartilage injury	63 (27)	54 (19)	117 (23)	0.037
MCL injury	6 (3)	8 (3)	14 (3)	0.846
**Surgical characteristics**				
Time to surgery (months), median (IQR)	6.0 (2.9–11.1)	4.4 (2.5–8.3)	5.1 (2.7–9.3)	0.013†
Surgical timing, n (%)				0.009
0–3 months	64 (28)	84 (30)	148 (29)	
3–6 months	53 (23)	95 (34)	148 (29)	
6–12 months	59 (25)	53 (19)	112 (22)	
12–24 months	56 (24)	47 (17)	103 (20)	
ACL-R graft type, n (%)				0.246
Hamstrings tendon	194 (84)	220 (79)	414 (81)	
Patellar tendon	20 (9)	37 (13)	57 (11)	
Quadriceps tendon	18 (8)	22 (8)	40 (8)	
Surgical procedures, n (%)				
Meniscus resection	78 (34)	72 (26)	150 (29)	0.053
Meniscus suture	60 (26)	62 (22)	122 (24)	0.337
Cartilage surgery	23 (10)	12 (4)	35 (7)	0.120

Statistical method: χ2 test.

Other sports: gymnastics, tennis/squash/padel, volleyball, motorsport, lacrosse, bandy, athletics, figure skating, orientation.

* Independent-samples t test.

† Mann-Whitney U test.

ACL, anterior cruciate ligament; ACL-R, ACL reconstruction; IQR, interquartile range; MCL, medial collateral ligament; n (%), number (per cent); SD, standard deviation.

Non-response analysis revealed significant differences in both sex and age distributions between included (n=748) and excluded (n=936) individuals, regardless of TAS level. Among included participants, 41% were male and 59% female, compared with 66% male and 34% female among those excluded (p<0.001). The mean age was lower in the included group than in the excluded group (25.4 years vs 26.5 years; p<0.001). There were no apparent differences in the type of activity at the time of injury (contact sports, non-contact sports, other), ACL-R graft choice or the prevalence of associated injuries between the included and non-included groups.

Baseline characteristics were similar between participants included in the multivariable analysis and those excluded due to missing ACL-RSI data (n=21), with no clinically meaningful differences in age, sex, preinjury activity level or sporting level (online supplemental table 1).

### Associated injuries and surgical characteristics

In total, 154 (30%) medial meniscus injuries, 187 (37%) lateral meniscus injuries and 117 (23%) cartilage injuries were identified, with a higher prevalence among males. Medial collateral ligament (MCL) injuries were infrequent, occurring in 14 cases (3%). The median time from injury to ACL-R was 5.1 months (IQR 2.7–9.3), and was slightly longer for males compared with females (6.0 months vs 4.4 months, p=0.013). Meniscal resection was performed in 150 participants (29%), and meniscal suturing in 122 patients (24%).

### Return to sport rates and functional outcomes

The mean time to RTS TAS≥7 was 12.3 months (SD 4.7), and the mean time to RTS the preinjury TAS level was 12.7 months (SD 4.9) across the cohort, without any significant differences between males and females (table 2).

**Table 2:** Return to sport rates to TAS≥7 and to preinjury TAS level within 12 months and 24 months and mean time to return to sports after primary ACL-R

	Male (n=232)	Female (n=279)	Total (n=511)	RR (95% CI)	P value
Time to RTS, mean (SD)					
Return to participation in TAS≥7	10.0 (5.1)	10.4 (5.0)	10.2 (5.1)	–	0.499*
Return to sport in TAS≥7	11.9 (4.7)	12.6 (4.5)	12.3 (4.7)	–	0.156*
Return to preinjury TAS level	12.2 (4.9)	13.1 (4.9)	12.7 (4.9)	–	0.148*
Return to participation in TAS≥7, n (%)					
At 12 months	135 (58)	153 (55)	288 (56)	0.94 (0.81 to 1.10)	0.446
At 24 months	171 (74)	195 (70)	366 (72)	0.95 (0.85 to 1.06)	0.338
Return to sport in TAS≥7, n (%)					
At 12 months	103 (44)	103 (37)	206 (40)	0.83 (0.67 to 1.03)	0.086
At 24 months	147 (63)	168 (60)	315 (62)	0.95 (0.83 to 1.09)	0.465
Return to preinjury TAS level, n (%)					
At 12 months	80 (35)	89 (32)	169 (33)	0.93 (0.72 to 1.18)	0.536
At 24 months	113 (49)	148 (53)	261 (51)	1.09 (0.92 to 1.29)	0.331

Statistical method: Log-binomial regression.

* Independent-samples t test.

ACL-R, anterior cruciate ligament reconstruction; RR, relative risk; RTS, return to sports; TAS, Tegner Activity Scale.

At 12 months, only 40% (n=206) had achieved RTS TAS≥7, and 33% (n=169) had returned to the preinjury TAS level after ACL-R. Overall, 62% of patients (n=315) achieved an RTS TAS≥7 after ACL-R, and 51% of participants (n=261) returned to their preinjury TAS level within 24 months. No significant differences between the sexes were found (table 2).

RTS rates and timing across sporting levels are shown in online supplemental table 2. No significant differences between sexes were observed across various sporting levels, with a tendency for more pronounced differences at lower divisions.

PROMs at 12 and 24 months demonstrated significantly higher functional scores among individuals who achieved RTS (defined as TAS≥7) than among those who did not achieve this threshold at any point during the study (online supplemental table 3).

### Survival analysis and predictors of returning to sports

Time points for RTS≥7 TAS and RTS preinjury TAS level (right) are graphically shown in a Kaplan-Meier analysis in figure 2A and B, with the number of censored per time point shown.

**Figure 2:**
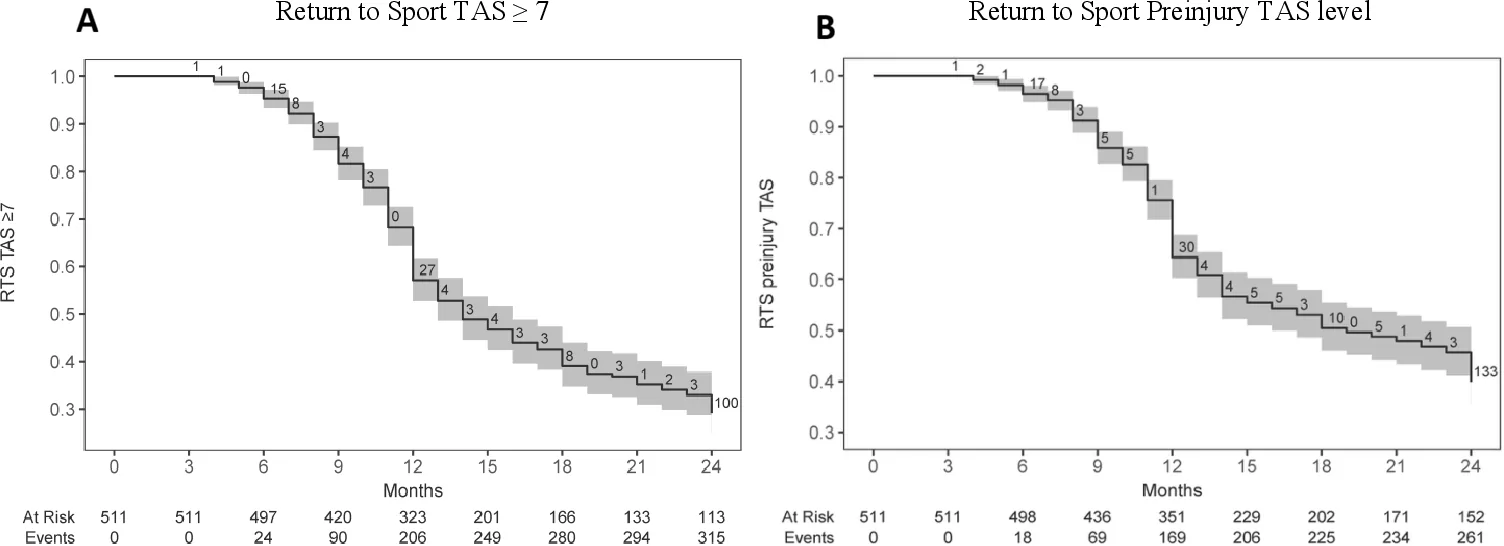
Kaplan-Meier analysis of the cumulative probability of returning to sport TAS≥7 (left), and of returning to sport to the preinjury TAS level (right). Censored participants are depicted in the curve as numbers at the different time points. RTS, return to sports; TAS, Tegner Activity Scale.

The multivariate Cox regression analysis (table 3) showed that females had a lower hazard of achieving RTS TAS≥7 (HR 0.71; 95% CI 0.56 to 0.90; p=0.005) than males. For each additional year of age, the hazard of RTS TAS≥7 decreased by 2% (HR 0.98; 95% CI 0.96 to 1.00; p=0.045), and the hazard of RTS preinjury TAS level decreased by 3% (HR 0.97; 95% CI 0.95 to 0.99; p=0.003).

**Table 3:** Independent predictors of RTS TAS≥7 and RTS preinjury TAS level after primary ACL-R*

	RTS TAS≥7		RTS preinjury TAS level	
	HR (95% CI)	p value	HR (95% CI)	P value
Sex				
Male	1 (ref)		1 (ref)	
Female	0.71 (0.56 to 0.90)	0.005	0.83 (0.64 to 1.08)	0.170
Age	0.98 (0.96 to 1.00)	0.045	0.97 (0.95 to 0.99)	0.003
Sporting level prior to ACL injury				
Elite	1 (ref)		1 (ref)	
Competitive	0.69 (0.53 to 0.90)	0.006	0.87 (0.65 to 1.16)	0.332
Recreational	0.32 (0.18 to 0.56)	<0.001	0.51 (0.27 to 0.94)	0.032
Surgical timing				
0–3 months	1 (ref)		1 (ref)	
3–6 months	1.19 (0.89 to 1.59)	0.238	1.07 (0.78 to 1.46)	0.693
6–12 months	0.91 (0.65 to 1.25)	0.548	0.90 (0.63 to 1.28)	0.552
12–24 months	0.57 (0.39 to 0.84)	0.005	0.48 (0.31 to 0.75)	0.001
Meniscus injury	0.84 (0.66 to 1.06)	0.140	0.73 (0.56 to 0.94)	0.016
Cartilage injury	0.93 (0.70 to 1.25)	0.629	0.93 (0.67 to 1.29)	0.656
MCL injury	0.40 (0.15 to 1.07)	0.069	0.37 (0.12 to 1.15)	0.086
ACL-RSI>4.2 at 3 months	1.72 (1.33 to 2.23)	<0.001	1.70 (1.28 to 2.25)	<0.001

Statistical method: Multivariable Cox proportional hazards regression.

* Multivariable regression based on 490 individuals; 21 observations were excluded due to missing ACL-RSI at 3 months.

ACL, anterior cruciate ligament; ACL-R, ACL reconstruction; ACL-RSI, ACL-Return to Sports after Injury score; HR, hazard ratio; MCL, medial collateral ligament; RTS, return to sport; TAS, Tegner Activity Scale.

Participants involved in competitive sports level had a lower hazard of RTS TAS≥7 compared with those involved in elite sports level (HR 0.69; 95% CI 0.53 to 0.90; p=0.006) and those engaged in recreational sports had an even lower hazard of RTS TAS≥7 (HR 0.32; 95% CI 0.18 to 0.56; p<0.001). Patients who underwent surgery more than 12 months after the injury had a lower hazard of RTS TAS≥7 (HR 0.57; 95% CI 0.39 to 0.84; p=0.005), and an even lower hazard of returning to the preinjury sports level (HR 0.48; 95% CI 0.31 to 0.75; p=0.001).

Meniscus injury had a negative effect on RTS preinjury TAS level (HR 0.73; 95% CI 0.56 to 0.94; p=0.016). The presence of an MCL injury showed a tendency toward a lower hazard of achieving RTS TAS≥7 (HR 0.40; 95% CI 0.15 to 1.07; p=0.069) and RTS preinjury TAS level (HR 0.37; 95% CI 0.12 to 1.15; p=0.086), but these findings were not significant. Psychological readiness at 3 months, as measured by the ACL-RSI score >4.2, was a strong predictor of successful RTS for both RTS TAS≥7 (HR 1.72; 95% CI 1.33 to 2.23; p<0.001) and RTS preinjury TAS level (HR 1.70; 95% CI 1.28 to 2.25; p<0.001).

### Second ACL injury

There were no differences in the rate of graft rerupture or failure between males and females (5.6% vs 5.7%; relative risk (RR) 1.02; 95% CI 0.50 to 2.08; p=0.949) within 24 months (table 4). Similarly, there was no difference in the occurrence of contralateral ACL injury (CACL) or CACL reconstruction between sexes (3.4% vs 3.6%; RR 1.04; 95% CI 0.42 to 2.59; p=0.934) during this time period.

**Table 4:** Risk of second ACL injury/surgery following primary ACL-R within 2 years per RTS TAS≥7 status and sex

	Males (n=232)	Females (n=279)	Total (n=511)
Graft rupture/failure			
No RTS≥TAS 7, n (%)	5 (5.9)	6 (5.4)	11 (5.6)
RTS≥TAS 7, n (%)	8 (5.4)*	10 (6.0)	18 (5.7)
RR (95% CI)†	1.08 (0.37 to 3.20)	0.91 (0.34 to 2.43)	0.98 (0.47 to 2.04)
P value	0.888	0.848	0.961
Total, n (%)	13 (5.6)*	16 (5.7)	29 (5.7)
RR (95% CI) (males vs females)	1 (ref)	1.02 (0.50 to 2.08)	
P value		0.949	
Revision ACL-R			
No RTS≥TAS 7, n (%)	5 (5.9)	6 (5.4)	11 (5.6)
RTS≥TAS 7, n (%)	7 (4.8)	8 (4.8)	15 (4.8)
RR (95% CI)†	1.24 (0.41 to 3.77)	1.14 (0.41 to 3.18)	1.18 (0.55 to 2.51)
P value	0.711	0.810	0.671
Total, n (%)	12 (5.2)	14 (5.0)	26 (5.1)
RR (95% CI) (males vs females)	1 (ref)	0.97 (0.46 to 2.06)	
P value		0.937	
CACL injury/CACL-R			
No RTS≥TAS 7, n (%)	1 (1.2)	3 (2.7)	4 (2.0)
RTS≥TAS 7, n (%)	7 (4.8)	7 (4.2)	14 (4.4)
RR (95% CI)†	0.25 (0.03 to 1.97)	0.65 (0.17 to 2.46)	0.46 (0.15 to 1.38)
P value	0.187	0.524	0.164
Total, n (%)	8 (3.4)	10 (3.6)	18 (3.5)
RR (95% CI) (males vs females)	1 (ref)	1.04 (0.42 to 2.59)	
P value		0.934	

Statistical method: Log-binomial regression.

* Includes one partial rupture of graft not leading to revision.

† RTS≥TAS 7 as reference.

ACL, anterior cruciate ligament; ACL-R, ACL reconstruction; CACL, contralateral anterior cruciate ligament; CACL-R, CACL reconstruction; n (%), number (per cent); RR, relative risk; RTS, return to sports; TAS, Tegner Activity Scale.

Likewise, when comparing the RTS TAS≥7 status (yes/no) for males, females or overall, no differences in the risk of graft rupture or contralateral ACL-R injury were observed.

## Discussion

The main finding of this longitudinal registry-based study was that the overall rates of RTS TAS≥7 and RTS preinjury TAS level were only 62% and 51%, respectively. Notably, the mean time for RTS TAS≥7 was 12.3 months, while the mean time for RTS preinjury TAS level was 12.7 months, reflecting a more modern and cautious RTS approach. Athletes achieving RTS TAS≥7 had better PROMs at both 12 and 24 months, suggesting a strong association with better functional recovery. Multivariable survival analysis showed that female sex, older age, non-elite sporting level and delayed ACL-R were associated with a lower likelihood of achieving RTS TAS≥7. Meanwhile, psychological readiness at 3 months, measured by ACL-RSI>4.2, emerged as an important and potentially modifiable factor associated with successful RTS outcomes after ACL-R.

### Return to sport rates and timing

The RTS rates in the current study are consistent with prior systematic reviews, suggesting persistent barriers to RTS after ACL-R despite years of advances in ACL research.^4 5 22^ A slight majority of athletes achieve RTS TAS≥7 after ACL-R, though only half reach preinjury TAS levels. Furthermore, only 72% returned to participation in a TAS≥7 sport, highlighting that a substantial proportion continue to experience limitations returning to high-demand knee activities post-ACL-R. But should we strive to improve these RTS rates, or is a lower RTS rate acceptable in the long run? The clinical importance of achieving RTS TAS≥7 following ACL-R is supported by our secondary analyses of PROMs and their associations with RTS TAS≥7, aligning with prior research.^23–25^ Moreover, the present study found no increased risk of secondary ACL injury among those achieving RTS TAS≥7, indicating that, for many, failing to reach an RTS TAS≥7 may not provide a protective benefit.

The mean time to achieve a RTS TAS≥7 was 12.3 months, with only 40% of participants reaching this threshold by 12 months. The optimal timing of RTS following ACL-R remains a complex and debated issue. Although evidence suggests that returning before 9 months post-ACL-R carries a substantially higher risk of re-injury,^26^ RTS outcomes appear to depend more on how athletes return than on when they return, indicating that less emphasis should be placed on time alone.^27^ One reason for the somewhat delayed RTS in the current study could be that the rehabilitation in this cohort after ACL-R was primarily goal-based rather than time-based.^27^ Our findings could also indicate that the RTS definition requiring full participation (with or without problems) used in the current study is somewhat stringent and may be challenging to accomplish.

### Predictors of return to sport

Contrary to expectations, this study found no significant differences in RTS rates between male and female athletes. The composition of this cohort, with a higher percentage of elite female athletes compared with males (31% vs 23%), may contribute to this finding. In fact, the male and female elite athletes in this cohort exhibit minimal differences in RTS TAS≥7 rates (76% vs 74%; RR 0.98; 95% CI 0.81 to 1.20; p=0.858), consistent with the current literature. Elite female athletes performing at the highest level of sport do not consistently demonstrate impaired RTS rates compared with males since studies have reported RTS rates as high as 79% to 82% in elite female athletes,^28 29^ which are comparable to the 83% RTS rates presented by Lai *et al* in a systematic review and meta-analysis primarily including studies with elite male athletes.^5^ Our multivariate Cox regression analysis, however, indicated that female sex was associated with a reduced probability of attaining RTS TAS≥7, suggesting that factors beyond sex may influence RTS rates among athletes. The discrepancy between unadjusted and adjusted analyses for sex likely reflects differences in baseline characteristics, particularly age, sporting level, associated injuries and surgical timing, highlighting the importance of multivariable adjustment when evaluating RTS outcomes.

These findings suggest that sex may not act as an isolated determinant of RTS, but rather reflects the influence of interacting factors such as sporting level, psychological readiness and access to support. Providing adequate psychological and social support may be a key component in helping these athletes manage the RTS process, as recovery after ACL injury often involves negative thoughts, emotional distress and frustration, which can hinder the rehabilitation process and preparation to RTS.^30 31^ Non-elite female athletes may lack the support needed during rehabilitation to overcome knee-related and psychological issues, such as fear of reinjury, potentially influencing these results.^6^ Interestingly, although the relatively low number of second ACL injuries, no sex-based differences in ACL reinjury rates were observed, aligning with prior research,^32^ indicating that female athletes should not be advised to expect a higher risk of reinjury on RTS than their male counterparts.

Beyond the differences between male and female athletes, older age, non-elite sporting level and delayed surgery were associated with a lower likelihood of achieving RTS TAS≥7. The impact of older age may reflect how it serves as a proxy for other priorities in life, such as family and employment commitment, which may be more important than RTS for older individuals.^4^ Another possible explanation is that younger athletes are less concerned about the potential risk of RTS, especially early RTS, and may prematurely return to high-demanding pivoting activities,^33^ despite the fact that young age and RTS are well-established risk factors for second ACL injury.^34^

Likewise, elite athletes in our study exhibit a higher RTS rate following primary ACL-R compared with those participating at competitive and recreational levels (75% vs 60% vs 33%, online supplemental table 2). Non-elite athletes may lack traits, such as greater athletic skill, greater value to a team, greater experience in overcoming injuries, financial and contractual commitments and a more positive response to injuries, that have been suggested to explain the higher RTS rates in elite athletes.^5^

Furthermore, delayed surgery may signal a different type of athlete compared with the elite athletes who often undergo surgery relatively early due to contractual obligations and performance demands, leading to a higher RTS rate than non-elite athletes.^35^ They may also be more cautious and have adapted to the ACL-injured knee, thereby more consciously reducing exposure to risk situations, for example, by avoiding RTS TAS≥7.^36 37^ However, these findings should be interpreted with caution, as they may reflect differences in athlete characteristics rather than a causal effect. Another alternative explanation for reduced RTS rates among individuals undergoing delayed ACL-R is the increased prevalence of associated knee injuries, such as meniscal and cartilage injuries, and increased knee laxity, which have been observed to be more common in this population.^20^ The observed association between meniscal injury and reduced likelihood of returning to preinjury TAS level of sports may reflect greater joint pathology and persistent knee-related symptoms that limit the ability to resume high-level sport.

### The role of early psychological readiness

While the above-mentioned factors are key factors associated with RTS, none are potentially modifiable after ACL-R. It is therefore of special interest to assess how the ACL-RSI can serve as an early predictor of RTS, as it measures an athlete’s psychological readiness^38^ which may be a modifiable factor after surgery. Compared with preinjury ACL-RSI scores, the greatest increase in ACL-RSI scores occurs in the period 3–6 months, with very little improvement thereafter.^39^

The current study adds to the limited body of research assessing the predictive value of early psychological readiness (ACL-RSI at 3 months) using a validated threshold at 4.2 (42 in the international ACL-RSI).^40^ Dichotomisation of ACL-RSI was performed to facilitate clinical interpretation and identification of individuals at risk of not returning to sport, in line with previous literature.^40^ Different cutoffs at different time points in the rehabilitation process have been used to assess RTS with good predictive ability. However, these studies assess ACL-RSI late in the rehabilitation process (between 6 and 9 months),^21 40 41^ except for one study evaluating ACL-RSI at 4 months.^42^ Our results indicate that ACL-RSI at 3 months could be used to assess individuals at risk of not RTS. Individuals who achieve a score higher than 4.2 on the ACL-RSI at 3 months will have a 72% higher likelihood of attaining RTS and 70% higher likelihood of returning to preinjury TAS level. However, these findings reflect associations and do not imply that modifying ACL-RSI will necessarily improve RTS outcomes. Interestingly, the ACL-RSI score at 12 months was significantly higher for those reaching RTS TAS≥7 than for those not achieving RTS TAS≥7.

### Clinical implications

The present study underscores the importance of adequately preparing athletes for realistic RTS rates and timelines following ACL-R. Athletes should be counselled not to expect to return to their respective sports within a predetermined timeframe. Additionally, athletes should be adequately prepared for the challenging nature of postoperative rehabilitation, as only 51% of athletes regain their preinjury level, as previously reported.^4 22^ Furthermore, clinicians should strive to assist athletes in returning to their desired sporting activity, as our results show this is associated with improved outcomes, aligning with the current literature.^23–25^ Clinicians should also consider identifying athletes with low psychological readiness early in the rehabilitation process, as this could provide a window for targeted psychological and physical interventions in their rehabilitation. Specifically, female, older-aged and non-elite athletes, identified as factors associated with a lower likelihood of RTS in this study, might benefit from this, as it may be difficult for them to seek and receive this support. Finally, athletes undergoing delayed ACL-R should also be informed of the increased challenges and potential difficulty in achieving a successful RTS when discussing the expected outcome after surgery.

### Strengths and limitations

One of the main strengths of the study is the goal-oriented definition of RTS, which focused on full participation in a specific activity level (classified using the TAS), with or without knee symptoms. This contrasts with the majority of studies included in a recent systematic review on RTS definitions, where RTS was typically defined as participation in a match, event or other competitive setting.^9^ Another key strength of this study is its prospective, longitudinal design with monthly follow-up, which improves the precision with which RTS timing is captured and minimises recall bias. It is also worth noticing that we achieved a high response rate for our primary outcome question regarding RTS, with only 80 individuals being lost to follow-up before potential RTS, giving us a 94% response rate at 12 months and 84% at 24 months, thereby reducing the likelihood of attrition bias in the selected TAS≥7 population (figure 1).

However, several limitations should also be considered when interpreting the current findings. The first important limitation is the lack of generalisability to the general population, as our study population differed significantly in sex and age from the population that was not interested in participating in our study. This introduces a potential risk of selection bias to the cohort, and this could have several reasons. First, older individuals are probably less motivated to RTS and therefore less interested in participating in a study evaluating this specific outcome. Second, females are more likely to respond to surveys and participate in studies like ours. Following non-response in the SNKLR, older age and female sex have been reported to improve overall response rates to KOOS (Knee injury and Osteoarthritis Outcome Score).^43^ However, this limited generalisability is aligned with the study’s objective, which was to specifically investigate return to sport in athletes with a preinjury TAS≥7. The aim was not to represent the general ACL population but rather to provide focused insight into RTS outcomes in highly active individuals. Moreover, the study’s nationwide recruitment, encompassing a wide range of clinics and rehabilitation settings, enhances the applicability of its findings to active athletes in clinical practice across Sweden. However, it is also important to note that age range was restricted to athletes aged 16–40 years to focus on skeletally mature individuals for whom return to high-level sport is clinically relevant; therefore, the results may not be generalisable to skeletally immature patients or to older individuals, who represent distinct clinical populations with different activity profiles and RTS expectations.

A third limitation is that although the use of a TAS≥7 provides a validated and standardised definition of high-level sport participation, the scale primarily reflects overall activity level rather than the specific demands associated with different sports or competitive contexts.^14^ Factors such as playing position, training frequency and volume, match exposure and level of competition are not captured by the Tegner scale, yet may substantially influence rehabilitation demands, injury risk and the likelihood of successful return to sport.

The fourth limitation is that standardised rehabilitation protocols were not used, as expected in a nationwide cohort. Although information on rehabilitation frequency was collected, detailed rehabilitation data and objective RTS test results were unavailable. Therefore, variability in rehabilitation and functional recovery may have influenced RTS outcomes but could not be accounted for in the analyses.

The fifth limitation is the potential impact of the COVID-19 pandemic, which overlapped with the study period and may have influenced access to rehabilitation, training opportunities and the RTS timeline. Although Sweden implemented relatively few restrictions compared with other countries, and RTS definitions and follow-up were standardised, external restrictions such as sporting event cancellations could have affected outcomes independently of surgical or patient-related factors. Future studies using this cohort will investigate the specific effects of the pandemic on RTS patterns and recovery trajectories. Finally, the relatively low number of secondary ACL injuries limited statistical power for these analyses and may have reduced the ability to detect associations between RTS and subsequent ACL injury outcomes. Consequently, these findings should be interpreted with caution.

## Conclusion

Our results showed overall RTS rates comparable to the current literature, where two-thirds achieved RTS TAS≥7 within 24 months, and only half of the individuals returned to preinjury TAS levels in sports. The mean time to RTS TAS≥7 was slightly beyond 12 months, possibly suggesting a tendency toward goal-based rehabilitation protocols among practitioners. Although crude RTS rates were similar between sexes, multivariable analysis showed that female sex, older age, lower sporting level and longer time from injury to surgery were associated with a lower likelihood of achieving RTS TAS≥7. Psychological readiness at 3 months (ACL-RSI>4.2) was strongly associated with RTS TAS≥7 and RTS preinjury TAS level and may represent an important target for future studies. These findings reinforce the importance of psychological readiness in relation as a vital component in achieving successful RTS outcomes. It should be followed more frequently after ACL-R to better identify who may need specific psychological support early in rehabilitation to reach RTS.

## Supplementary material

10.1136/bjsports-2025-111497 online supplemental file 1

## Data Availability

No data are available.
