# Supplementary material for: Return to sport after primary anterior cruciate ligament reconstruction in athletes with a preinjury Tegner Activity Scale ≥7: a longitudinal, nationwide, registry-based cohort study
Source: Br J Sports Med. 2026 Oct 8;60(20):e111497. doi: 10.1136/bjsports-2025-111497 (PMC13691718; doi:10.1136/bjsports-2025-111497)
Supplement: Supplementary file 1 [file bjsports-60-20-s001.pdf]

Supplement Material

Supplement Table 1. Comparison of participants included and excluded from the multivariable analyses due to missing ACL-RSI data

Characteristic	Included (n = 490)	Excluded (n = 21)	p-value
**Age (years), mean (SD)***	24.0 (6.5)	22.2 (5.0)	0.205
**Female sex, n (%)**	269 (55)	10 (48)	0.655
**Preinjury Tegner Activity Scale**			0.933
Tegner 7, n (%)	114 (23)	5 (24)	
Tegner 8, n (%)	98 (20)	3 (14)	
Tegner 9, n (%)	216 (44)	10 (48)	
Tegner 10, n (%)	62 (13)	3 (14)	
**Sporting level**			0.842
Elite, n (%)	132 (27)	6 (29)	
Competitive, n (%)	311 (64)	14 (67)	
Recreational, n (%)	47 (10)	1 (5)	

Statistical method: *Student's t-test for continuous variables; Chi-squared for categorical variables.

Supplement table 2. Return to sport rates to TAS ≥7 and to preinjury TAS level within 12 months and 24 months and mean time to return to sports after primary ACL-R per Sporting level

	Male	Female	Total	RR (95% CI)	p-value
**Elite athletes, n**	53	85	138		
**Return to sport in TAS ≥7**					
Time to RTS, months, mean (SD)	11.0 (3.9)	12.5 (4.2)	11.9 (4.2)	–	0.081*
At 12 months, n (%)	32 (60)	41 (48)	73 (53)	0.80 (0.59–1.09)	0.156
At 24 months, n (%)	40 (76)	63 (74)	103 (75)	0.98 (0.81–1.20)	0.858
**Return to preinjury TAS level**					
Time to RTS, months, mean (SD)	11.8 (5.1)	13.1 (4.9)	12.6 (5.0)	–	0.282*
At 12 months, n (%)	22 (42)	34 (40)	56 (41)	0.96 (0.64–1.46)	0.860
At 24 months, n (%)	29 (55)	53 (62)	82 (59)	1.14 (0.85–1.53)	0.386
**Competitive athletes, n**	149	176	326		
**Return to sport in TAS ≥7**					
Time to RTS, months, mean (SD)	12.0 (5.0)	12.6 (4.4)	12.3 (4.7)	–	0.317*
At 12 months, n (%)	65 (44)	60 (34)	125 (39)	0.78 (0.59–1.03)	0.079
At 24 months, n (%)	96 (64)	100 (57)	196 (60)	0.88 (0.74–1.05)	0.160
**Return to preinjury TAS level**					
Time to RTS, months, mean (SD)	12.0 (4.5)	13.1 (4.8)	12.6 (4.7)	–	0.142*
At 12 months, n (%)	53 (36)	53 (30)	106 (33)	0.85 (0.62–1.16)	0.295
At 24 months, n (%)	74 (50)	91 (52)	165 (51)	1.04 (0.84–1.29)	0.714
**Recreational athletes, n**	30	18	48		
**Return to sport in TAS ≥7**					
Time to RTS, months, mean (SD)	14.1 (6.8)	13.8 (8.4)	14.0 (7.1)	–	0.942*
At 12 months, n (%)	6 (20)	2 (11)	8 (17)	0.56 (0.13–2.47)	0.439
At 24 months, n (%)	11 (37)	5 (28)	16 (33)	0.76 (0.31–1.83)	0.537
**Return to preinjury TAS level**					
Time to RTS, months, mean (SD)	14.6 (7.0)	13.3 (9.6)	14.2 (7.4)	–	0.773*
At 12 months, n (%)	5 (17)	2 (11)	7 (15)	0.67 (0.14–3.09)	0.604
At 24 months, n (%)	10 (33)	4 (22)	14 (29)	0.67 (0.25–1.82)	0.427

Statistical method: Log-binomial regression; * Independent-samples t test.

ACL-R, anterior cruciate ligament reconstruction; CI, confidence interval; RTS, return to sports; RR, relative risk; SD, standard deviation; TAS, Tegner Activity Scale; n (%), number (percent)

Supplement table 3. Patient reported outcome at 12- and 24-months follow-up between those reaching RTS TAS ≥7 and not reaching RTS TAS ≥7 within 24 months by sex

	No RTS TAS ≥7		RTS TAS ≥7		p-value
	**N (%)**	**Mean score (SD)**	**N (%)**	**Mean score (SD)**	
**Females**	111 (100)		168 (100)		
**IKDC-SKF**					
12 months	80 (72)	71.1 (14.8)	149 (89)	82.2 (11.7)	<0.001
24 months	51 (48)	74.3 (16.4)	114 (71)	85.9 (13.2)	<0.001
**SANE**					
12 months	79 (71)	75.6 (14.7)	147 (88)	83.6 (15.7)	<0.001
24 months	52 (49)	79.1 (16.8)	121 (75)	86.6 (16.9)	0.004
**ACL-RSI**					
12 months	80 (72)	4.9 (1.9)	149 (89)	6.5 (2.1)	<0.001
24 months	51 (48)	4.6 (2.1)	117 (73)	6.9 (2.3)	<0.001
**Males**	84 (100)		147 (100)		
**IKDC-SKF**					
12 months	52 (62)	70.2 (17.6)	121 (82)	85.2 (12.5)	<0.001
24 months	33 (42)	70.3 (15.5)	93 (69)	86.4 (13.6)	<0.001
**SANE**					
12 months	50 (60)	70.4 (20.8)	122 (83)	85.3 (15.2)	<0.001
24 months	33 (42)	72.6 (19.8)	95 (70)	88.0 (17.7)	<0.001
**ACL-RSI**					
12 months	52 (62)	4.8 (2.2)	119 (81)	6.9 (2.0)	<0.001
24 months	33 (42)	4.6 (2.2)	93 (69)	7.1 (2.2)	<0.001

Statistical method: Independent-samples t test.
ACL-RSI, Anterior Cruciate Ligament-Return to Sports Injury scale; IKDC-SKF, International Knee Documentation Committee-Subjective Knee Evaluation Form; RTS, return to sports; SANE, Single Assessment Numeric Evaluations; SD, standard deviation; TAS, Tegner Activity Scale; N (%), number (percent)

Supplement table 4. Associations between individual factors and return to sport to TAS ≥7 and to preinjury TAS level after primary ACL-R

	RTS TAS ≥7 (n=315)			RTS preinjury TAS level (n=261)		
	n (%) or mean (SD)	HR (95% CI)	p-value	n (%) or mean (SD)	HR (95% CI)	p-value
**Sex**						
Male	147 (63)	1 (ref)		113 (49)	1 (ref)	
Female	168 (60)	0.87 (0.70–1.08)	0.209	148 (53)	1.05 (0.82–1.35)	0.675
**Age at surgery**	23.4 (6.5)	0.97 (0.95–0.99)	<0.001	23 (6.2)	0.96 (0.94–0.97)	<0.001
**Sporting level prior to ACL injury**						
Elite	103 (75)	1 (ref)		82 (59)	1 (ref)	
Competitive	196 (60)	0.65 (0.51–0.82)	<0.001	165 (51)	0.71 (0.55–0.93)	0.013
Recreational	16 (33)	0.28 (0.17–0.48)	<0.001	14 (29)	0.35 (0.20–0.62)	<0.001
**Meniscus injury**						
No	148 (64)	1 (ref)		131 (56)	1 (ref)	
Yes	167 (60)	0.82 (0.66–1.03)	0.084	130 (47)	0.71 (0.56–0.90)	0.006
**Cartilage injury**						
No	251 (64)	1 (ref)		212 (54)	1 (ref)	
Yes	64 (55)	0.80 (0.61–1.06)	0.118	49 (42)	0.71 (0.52–0.98)	0.034
**MCL injury**						
No	311 (63)	1 (ref)		258 (52)	Ref.	
Yes	4 (29)	0.52 (0.20–1.41)	0.199	3 (21)	0.49 (0.16–1.53)	0.220
**Surgical timing, months (interval)**						
0–3 months	100 (68)	1 (ref)		86 (58)	1 (ref)	
3–6 months	103 (70)	1.11 (0.84–1.46)	0.467	86 (58)	0.99 (0.73–1.33)	0.928
6–12 months	66 (59)	0.80 (0.58–1.09)	0.154	56 (50)	0.80 (0.57–1.12)	0.191
12–24 months	46 (45)	0.46 (0.33–0.66)	<0.001	33 (32)	0.37 (0.25–0.55)	<0.001
**Meniscus resection**						
No	226 (63)	1 (ref)		192 (53)	1 (ref)	
Yes	89 (59)	0.90 (0.71–1.16)	0.419	69 (46)	0.80 (0.61–1.05)	0.107
**Meniscus suture**						
No	239 (61)	1 (ref)		200 (51)	1 (ref)	
Yes	76 (62)	0.90 (0.69–1.16)	0.420	61 (50)	0.87 (0.66–1.16)	0.351
**Cartilage Surgery**						
No	299 (63)	1 (ref)		251(53)	1 (ref)	
Yes	16 (46)	0.73 (0.44–1.21)	0.223	10 (29)	0.47 (0.25–0.89)	0.020
**ACL-R graft type**						
BPTB	33 (58)	1 (ref)		28 (49)	1 (ref)	
Hamstring	258 (62)	1.09 (0.76–1.57)	0.627	213 (51)	1.05 (0.71–1.56)	0.800
Quadriceps	24 (60)	0.94 (0.56–1.59)	0.817	20 (50)	0.93 (0.52–1.64)	0.791
**ACL-RSI at 3 months ***						
ACL-RSI <4.2	87 (48)	1 (ref)		71 (39)	1 (ref)	
ACL-RSI >4.2	213 (69)	1.84 (1.43–2.36)	<0.001	178 (58)	1.83 (1.39–2.40)	<0.001

Statistical method: Univariate Cox proportional hazards regression.
ACL-R, anterior cruciate ligament reconstruction; ACL-RSI, Anterior Cruciate Ligament-Return to Sports after Injury score; BPTB, bone-patellar tendon-bone; CI, confidence interval; HR, hazard ratio; MCL, medial collateral ligament; RTS, return to sport; TAS, Tegner Activity Scale; n (%), number (percent)
* 21 observations excluded due to missing data.
